# Emotion, attention and stress regulation as markers of resilience in male and female Israeli soldiers during the Israel–Hamas war

**DOI:** 10.1007/s00406-024-01948-z

**Published:** 2024-12-29

**Authors:** Rotem Cohen, Janne L. Punski-Hoogervorst, Inon Maoz, Batya Engel-Yeger, Lucian Tatsa-Laor, Avi Avital

**Affiliations:** 1 Behavioral Neurobiology Lab, Department of Occupational Therapy, Faculty of Social Welfare and Health Sciences, University of Haifa, 199 Aba Khoushy Ave., Mount Carmel, Haifa, Israel; 2 Medical Corps, Israel Defense Force, Tel Aviv, Israel

**Keywords:** Resilience, Emotional regulation, Attentional regulation, Auditory sustained attention test, Electrodermal activity, Hypervigilance

## Abstract

Psychological resilience is a key factor for societal and military stability when faced with terror attacks and/or war. The research presents physiological findings—obtained with the electrodermal activity (EDA) and Auditory Sustained Attention Test (ASAT)—on stress responses, attentional and emotion regulation abilities in 57 Israel Defense Force male and female combat soldiers during the ongoing Israel–Hamas war. In addition, it shows self-reported resilience scores and post traumatic symptomatology measured by questionnaires and explores the relationship between the subjective and objective data. Compared to male soldiers, female soldiers showed significantly higher hyperarousal symptoms yet showed a tendency to a significantly lower specific skin conductance response (on the EDA) to the first startle sound. Furthermore, the self-reported acute stress symptoms positively and significantly correlated with the physiological emotion regulation measured by startle responses, and negatively correlated with attentional regulation measured by the ASAT. The lack of gender differences in stress level, resilience and self-regulation abilities emphasizes the high capabilities of women combat soldiers, especially due to gender-related risks in combat. Relatively high scores of acute stress symptomology in the population of combat soldiers invite later screening and assessment for the prevention of post traumatic disorders in vulnerable individuals. The combination of physiological measures and questionnaires highlights possible report biases, and thus underscores the importance of combining these objective/subjective measures for adequate assessment of resilience and post traumatic symptomology.

## Introduction

On October 7th, 2023, a highly coordinated surprise attack of the Islamic militant group Hamas—designated as a terrorist group by many countries, including the United States and the European Union—caused a wave of terror attacks in Israel. Approximately 1400 people in the south of Israel, mostly civilians, were brutally murdered, and more than 240 people were taken hostage in Gaza. In response, Israel declared a war on Hamas, titled “Iron Swords”.

Although there is a long-standing conflict between Israel and Hamas, the recent wave of violence is regarded as exceptionally severe by the Israeli society. Among the reasons for this is the fact that it’s been the deadliest day in the nation’s history [1], as well as the terror acts being widely broadcasted on social media (coming from the terrorists that self-videotaped their horrifying acts), displaying unspeakable atrocities—including rape, torture, and murder of both soldiers and civilians—on a mass scale [2].

It has previously been established that the resilience of soldiers is affected by their personal relation to the war and terror events, the risk they are or might be exposed to; the effects on their family, friends, and community; and the interpretation of the events, including the historical meaning and societal appraisal [3]. The Israel Defense Force (IDF), as “the people's army” [4], is based on the notion of ‘Never Again’, relating to millennia of persecution of the Jewish people, culminating in the Holocaust [5]. The October 7th massacre echoes those events and feelings, as the attack was mainly aimed at unarmed and helpless civilians—including infants, children, and elder people—in their homes they regarded as their safe-haven. The perceived violation of the ‘Never Again’ principle is a source of motivation, reflected in the overwhelming response to the reserve soldier's emergency recruiting of 360,000 reservists—including many volunteers—in one of the largest mobilizations in Israel’s history [6]. However, the direct and severe threat to places, people and lives that are dear to the soldier is on the other hand a source of hypervigilance and distress, affecting current and future mental health.

Exposure to extreme events, that include a life threat or a violation of moral values, leads in a minority of the cases to later psychiatric disorders. A short-term consequence may be the experiencing of symptoms of Acute Stress Response (ASR), namely intrusive symptoms, negative mood, dissociation, avoidance, and hyperarousal. When these symptoms continue for over one month, a person might meet the diagnostic criteria for posttraumatic stress disorder (PTSD) [7]. On the other side of the mental balance, one finds psychological resilience. Resilience is the ability to maintain an emotional and cognitive equilibrium with continuation of the normal daily life functioning, despite exposure to potentially traumatic events (PTEs) [8]. When looking into resilience as an ability, it is commonly viewed as an amalgamation of external and internal factors [9]. Integral internal factors are emotion or affect regulation [10, 11] and attentional capacities [12–16]. Specifically for military personnel, additional important intrapersonal factors that have been indicated as relevant for resilience and personal competence are self-efficacy and positive self-perception [17]. Furthermore, the personality disposition of hardiness has been recorded as a mediator between service duration and self-reported PTSD symptoms. Hardiness is described as a positive attitude to stress, that combines a commitment to a mission and/or higher principles, the belief that hardship is challenging yet growth-promoting, and a sense of control and influence over one’s own life [18].

Resilience is usually measured through self-report measures, despite concerns with regards to their external validity [19, 20]. To achieve a comprehensive view of this pivotal variable, we used self-report in addition to physiological measures of arousal, attentional and emotional regulation. The latter were previously established as predictors of PTSD symptomology and anxiety, and therefore were considered as relevant parameters of stress resilience [21–24].

There is some psychological and neurophysiological evidence that resilience can manifest differently in men and women [25–28]. Moreover, differences in emotional awareness that point to a female advantage regarding resilience [29], may be reflected in self-reported measures, where men may report their feelings with lesser accuracy, but not in physiological ones. This enhanced emotional awareness might contribute to self-regulation strategies that might strengthen actual resilience in females [30].

This research aimed to investigate psychological and physiological measures of emotional, attentional and stress regulation of IDF soldiers in combat roles, mostly in their first few months of mandatory army service, considering their involvement in the war following the October 7th massacre. The male and female soldiers participating in the research were in basic combat training and other active-duty combat roles within the training base. We hypothesized that: Female soldiers will report lower resilience then males on some or all of the self-report measurements but will show better resilience on some or all of the physiological measurements.

## Methods

This cross-sectional study was conducted with active-duty soldiers from the IDF. Data was collected on October 18 and 19, 2023. The participating soldiers had all been called to report for duty in the days directly following the October 7th massacre and had thus not been on base during the actual massacre and the initial combats that ensued, although other soldiers from their units and battalions were involved in the fighting. All data was obtained on a base in the southern part of Israel, away from the active front near the Gaza border.

### Participants

The study population consisted of 57 anonymized combat soldiers (male and female) during early stages of their mandatory army service. Additional 6 participants did not complete their measurement due to technical difficulties and therefore their data was not analyzed. All participants came from varied socioeconomic backgrounds, ethnicities, and nativity status, which we could not investigate further due to military ethical restrictions. However, we were allowed to record socioeconomic information (see Table 1). Inclusion criteria: serving in a combat role and willing to participate in the study. Exclusion criteria were hearing problems, chronic diseases, or the use of illicit drugs.

**Table 1:** Socio-demographic characteristics and resilience scores

	Female soldiers (n = 39)	Male soldiers (n = 18)
Age in years	Mean = 18.67 (SD 0.73)	Mean = 18.72 (SD 1.02)
Duration of army service in months	Mean = 4.33 (SD 4.68)	Mean = 5.07 (SD 7.34) *p* = *0.65,* *95%* *CI* *[−* *3.983,* *2.508]*
Adaptability to army perceived as easy	n = 22 (56.41%)	n = 16 (88.89%) *p* = 0.015*, *95%* *CI* *[−* *0.585,* *−* *0.065]*
Army service perceived as emotional burden	n = 28 (71.79%)	n = 4 (22.22%) *p* < 0.001*, *95%* *CI* *[0.24,* *0.751]*
Socioeconomic status—family income	Below average—10 (~ 25.6%) Average—15 (~ 38.5%) Above average—13 (~ 33.3%) No answer—1 (~ 0.03%)	Below average—6 (~ 33.3%) Average—5 (~ 27.8%) Above average—7 (~ 38.9%)
CD-RISC—total score	Mean = 30.39 (SD 5.47)	Mean = 32.58 (SD 4.25) *p* = *0.134,* *95%* *CI* *[−* *5.059,* *0.691]*

*Indicating a significant result

### Physiological measures

#### Auditory Sustained Attention Test (ASAT)

The Auditory Sustained Attention Test (ASAT) is a physiological test that records the eyeblink reflex through the electromyography (EMG) activity of the orbicularis oculi muscle, following an auditory stimulus. The ASAT measures both emotion regulation by recording the response to strong startle auditory stimuli, as well as sustained attention through a neurological phenomenon called pre-pulse inhibition (PPI). Specifically, a weak acoustic pre-pulse inhibits the startle reaction to a subsequent stronger auditory stimulus [21, 22]. PPI is a quantitative measurement of central processing, specifically sensorimotor gating mechanisms. They are related to the attentional ability to focus on saliant stimuli, while adaptively filtering out irrelevant information [31].

**Fig. 1:**
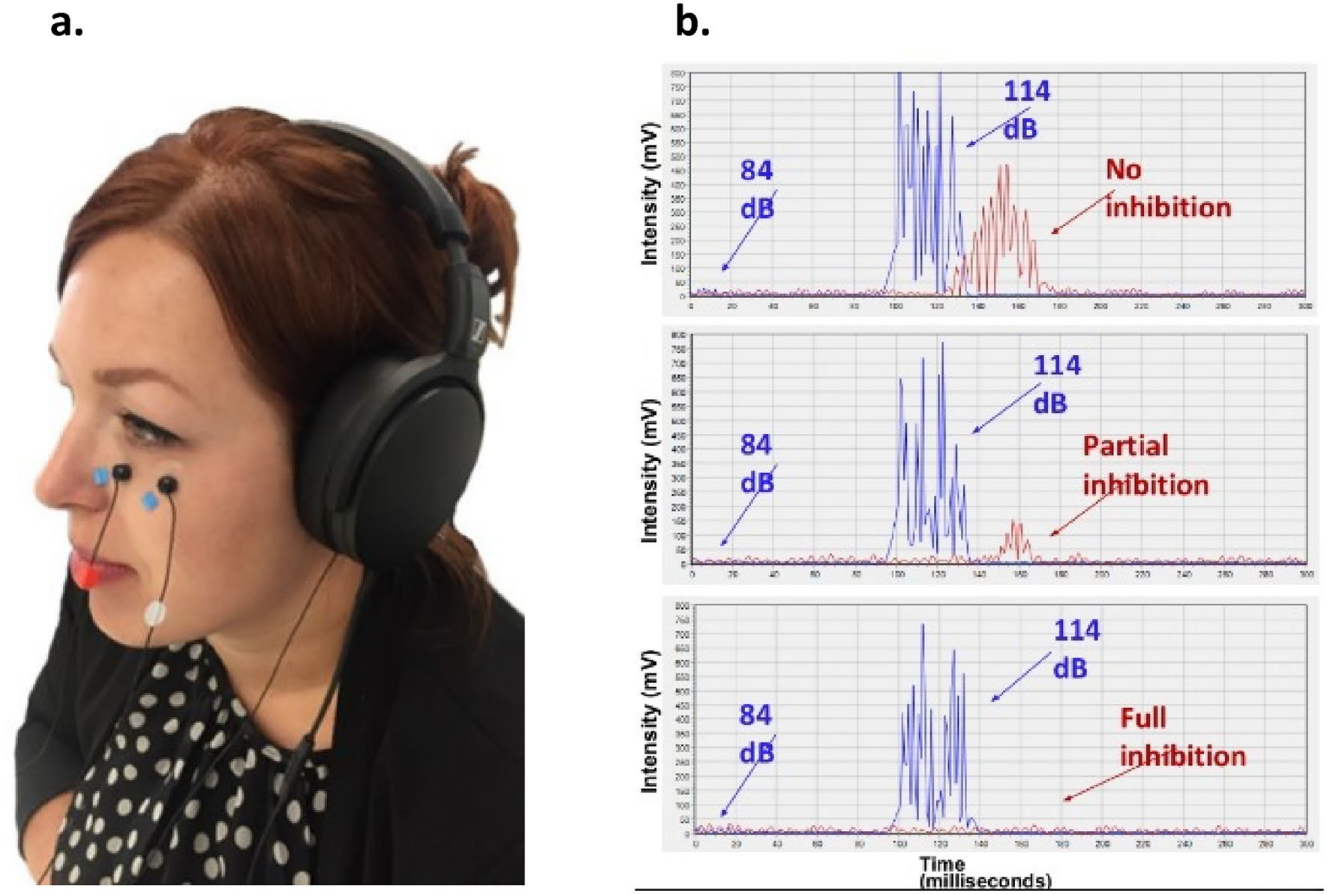
ASAT recording. **a** Placement of ASAT electrodes on the skin covering the left orbicularis oculi muscle. **b** Example of ASAT displaying three potential outcomes of pre-pulse abilities. Namely no-, partial- or full-inhibition of the eyeblink reflex. Displayed in blue is the auditory stimulus, and in red the recorded EMG response

A computerized startle response monitoring system (Mindtension ltd., Niram, Israel) was used to deliver acoustic startle stimuli via headphones while recording EMG of the eye-blink. Three electrodes with a recording area of 4 mm were attached to an adhesive disk (EL254 electrodes and ADD 204; Biopac Systems Inc., CA, USA) and filled in with a small amount of prepping gel (Signa Gel; Parker Laboratories Inc., Fairfield, NJ, USA). Two electrodes were placed approximately 1 cm below the pupil on the orbicularis oculi muscle (medial and lateral below to the outer corner), and the third (reference) electrode was placed on the mastoid bone (Fig. 1a).

The ASAT session started with a one-minute acclimatization period of 60 dB background noise level, which was delivered continuously throughout the session. The session then comprised of 60 pseudo-randomly delivered startling stimuli, at a 4-s average inter-trial interval (ranging from 2.5 to 5.5 s) with a total trial duration of six minutes. Thirty trials comprised of single 30 ms duration of 102-, 108- or 114-dB pulse intensities, 10 trials per intensity. As we previously reported [21, 22, 32, 33], these stimuli subserved as a measure of emotional regulation by the startle response patterns. To evaluate attentional regulation by the ASAT, 30 ‘pre-pulse' trials consisted of a weaker pre-pulse of 14-, 18- or 24-dB above background noise (i.e., 74-, 78- or 84 dB) 20 ms before a 114-dB startling stimulus [21, 22, 33].

Data were recorded with a 1 kHz sampling rate with a Band-Pass Filter of 10–300 Hz. For each audio stimulus, data analysis was performed on the first 300 ms time window (Fig. 1b). Using Mindtension software (Mindtension ltd., Nir Am, Israel), the following outcome measures were calculated:

- Median startle response amplitudes (in mV) to all 102-, 108- and 114-dB ‘pulse alone’ trials.
- Medians of ‘pre-pulse' trials to 74-, 78- or 84 dB per median of startle response to 114 dB in percentage (%).

#### Electrodermal activity (EDA)

The electrodermal activity (EDA) is a measurement of the changes in electrical skin conductance, which is related to physiological arousal through sweat gland activity [33]. Two 5-mm-diameter Ag–AgCl electrodes (Mindlife, Jerusalem, Israel) were applied to the fingertips of the second and third digits of the non-dominant hand and secured with a Velcro band. Electrodes were connected to a sensor and to an amplified receiver. An isolated skin conductance coupler (Mindlife, Jerusalem, Israel) applied a constant 0.5 V potential across the electrode pair.

The EDA was recorded simultaneously with the ASAT and therefore lasted six minutes. Data were recorded with ProRelax 5.1 by Mindlife (Jerusalem, Israel), using a sample rate of 10 Hz. After the test, data were exported and converted from arbitrary units to micro-siemens (μS). These converted data were then imported to Ledalab v3.4.9 (Graz, Austria; Kiel, Germany), powered on MATLAB 2023a (The MathWorks, Natick, MA, USA), to automatically extract the skin conductance levels using Continuous Decomposition Analysis with a peak amplification threshold of 0.05 µS [34]. For the phasic measures, the latency window for EDA onset was set at 1–4 s after the startle stimulus onset [35]. Finally, the data were imported into an Excel sheet for calculation of non-specific skin conductance responses (SCRs) as average per minute and analysis of the event-related SCR to the first startle response: the amplitude in µS, rise time in seconds, and 50% and 100% decay time in seconds.

### Questionnaires

All questionnaires were in Hebrew, in which all participants were fluent. First, a socio-demographic questionnaire was distributed to collect basic information regarding gender, age, army duration and subjective army experience. In addition, resilience, and ASR questionnaires were filled out:

1. *The* *Connor-Davidson* *Resilience* *Scale* *(CD-RISC)* This is an established self-report questionnaire of psychological or mental resilience, including hardiness, that consists of 10 items that are to be rated on a 5-point Likert scale (0 = not true at all to 4 = true nearly all the time). Higher scores reflect a greater personal resilience facet [36]. This 10-item unidimensional scale is considered to have good internal consistency (alpha = 0.85) and construct validity. External validity was shown through a combination of other subjective assessments, as the resilience scores significantly moderated the relationship between childhood maltreatment and current psychiatric symptoms [37].
2. *Stanford* *Acute* *Stress* *Reaction* *Questionnaire* *(SASRQ)* This questionnaire measures symptoms of acute stress response (ASR). It is therefore administered up to 30 days after a potentially traumatic event has taken place. It includes one open question that inquiries about the potentially traumatic event, two general assessment questions (“How disturbing was the event to you?” after the open question and “On how many days did you experience the symptoms of distress?” as the final question) on a 5-point Likert scale, and 30 questions that assess ASR symptoms on a 6-point Likert scale (with 0 = not experienced and 5 = very often experienced). The symptoms are divided into five subscales, namely: dissociation, re-experiencing, hyperarousal, avoidance, and imparity of functioning [38]. The SASRQ has consistently been shown as reliable and presenting convergent (r = 0.75 to 0.81), divergent and predictive validity of future PTSD diagnosis, in addition to high internal consistency (alpha = 0.79 to 0.95)[39].

### Procedure

Ethical approval was obtained from the IDF Helsinki committee (approval 2301–2023). Data were collected anonymously and in accordance with the World Medical Association Declaration of Helsinki. An army base in southern Israel was selected for the variety and availability of different combat soldiers. During the research days, after receiving a short explanation of the study's course and purposes, the soldiers who were interested in participating, signed an informed consent form. The measurements took place in a quiet and private room, where the ASAT and EDA were performed simultaneously, and the questionnaires were filled out digitally before or after the physiological measurements. A researcher was available to answer questions and concerns regarding any part of the research, and a mental health officer was available as a backup support.

### Statistical analysis

All data were imported to an Excel file and data analysis was then conducted in three stages: (1) descriptive statistics of the socio-demographic variables and various test outcomes were calculated for the female and male soldiers separately; (2) a between subjects' independent sample t-test was conducted for scales, and chi-square tests for nominal data; (3) a correlation analysis of the various test outcomes was conducted using Pearson’s correlation coefficient. Analyses were conducted using IBM SPSS Statistics for Windows, version 29.0.1.0 (IBM Corp, NY, USA). Results were considered significant if the p-value was < 0.05. The Bonferroni correction for statistical significance was applied in case of multiple comparisons.

## Results

**Fig. 2:**
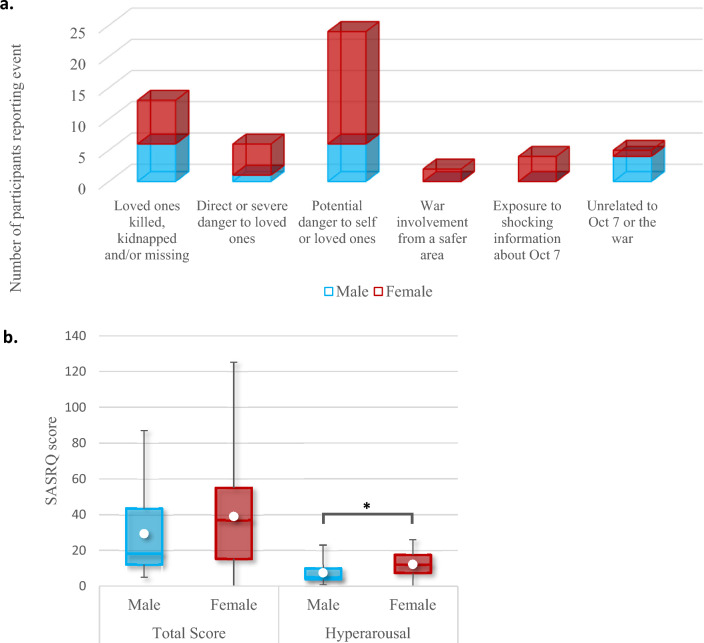
SASRAQ outcome measures. **a** A quantification of the qualitative responses to most stressful event. **b** Boxplots with additional means reflecting the SASRQ total scores and hyperarousal subscale scores of the male and female soldiers. Although the total score was not significantly different between the groups, females did report a significantly higher level of hyperarousal symptoms [p = 0.011, 95% CI (1.111, 8.258)]

Soldiers were asked—as part of the SASRQ—which event they regarded as most stressful since the start of the Israel–Hamas war. Answers included the following examples: “When I found out that my battalion members were in an encounter with terrorists, and I knew there were wounded and dead. But I didn’t know who and if I knew them or not.’’; “Murdered friends.”; “Death of relatives”. Most soldiers (n = 24; 42.11%) described an event related to potential danger to themselves or loved ones, followed by reports of loved ones being killed/kidnapped/missing (n = 13; 22.81%) (Fig. 2a). The quantitative data of the SASRQ showed a significant difference between the male and female combat soldiers on self-reported hyperarousal symptoms [mean male = 7.53 (SD 5.56), mean female = 12.21 (SD 6.69), p = 0.011, 95% CI (1.111, 8.258)], but not on the total score [mean male = 29.42 (SD 22.50), mean female = 39.05 (SD 27.00), p = 0.186, 95% CI (-4.791, 24.054)] or other subscales. See Fig. 2b for the boxplot of the SASRQ total scores and hyperarousal subscale scores.

### ASAT

**Fig. 3:**
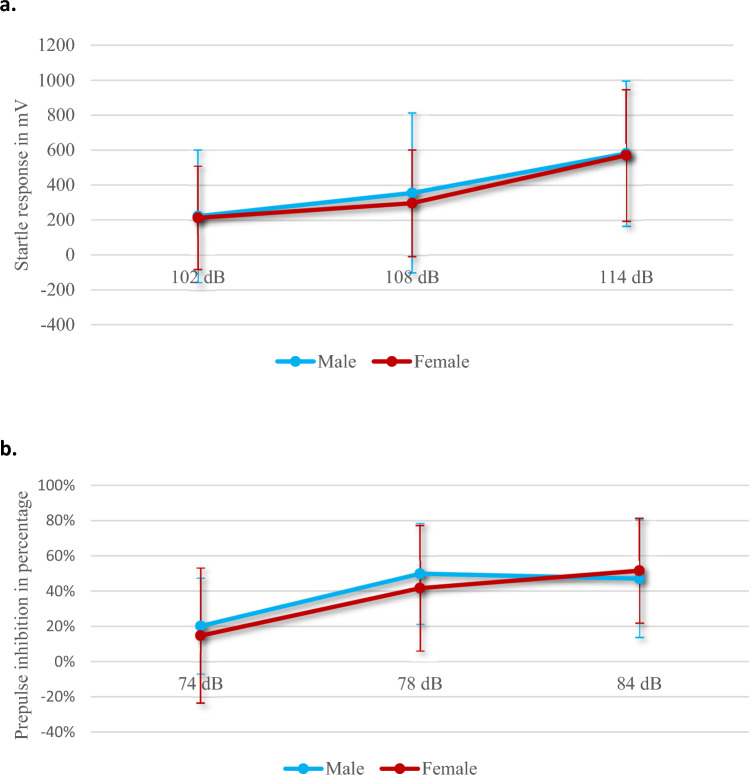
Emotional and attentional regulation. **a** Mean and standard deviation of the startle responses in millivolt per startle level (102, 108 and 114 dB) for both female and male soldiers, showing similar dose-related responses, thus indicating intact emotional regulation. **b** Mean and standard deviation of the pre-pulse inhibition in percentage per pre-pulse level (74, 78 and 84 dB), showing similar intact attention regulation for both female and male soldiers

**Fig. 4:**
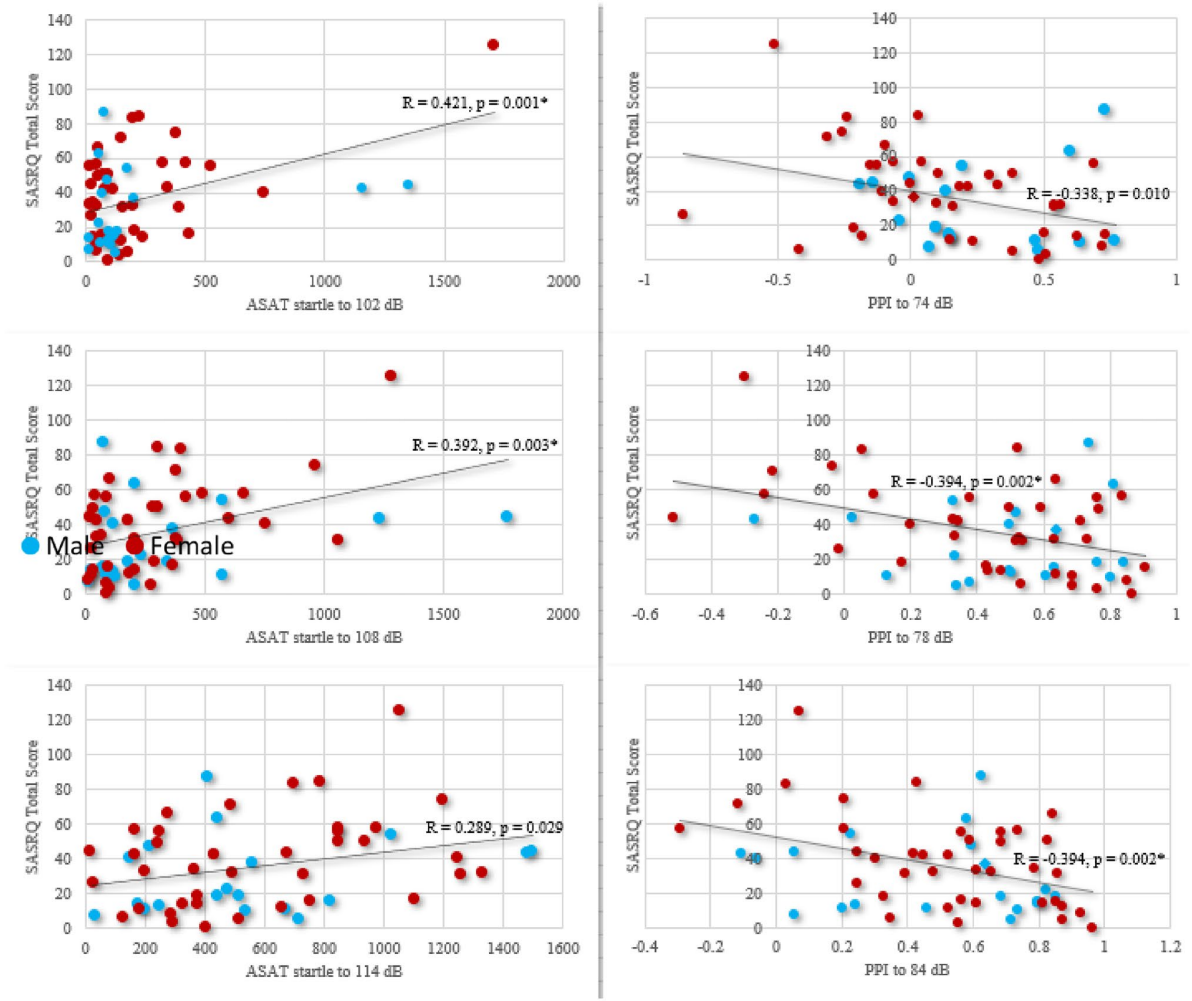
Correlation calculation of the SASRQ total scores in relation to emotional regulation (startle responses to 102-, 108- and 114-dB), and attentional regulation (pre-pulse inhibition with 74-, 78- and 84-dB pre-pulse intensities) for the male (blue) and female (red) soldiers. Results show significant and positive correlations between the SASRQ scores and emotional regulation (at 102 and 108 dB), while negatively correlating with the attentional regulation (at 78 and 84 dB). The alpha level for a significant correlation was adjusted to p = 0.008, following Bonferroni correction

The ASAT scores of both gender groups showed normal dose-related startle responses (Fig. 3), with no significant differences between the groups. However, correlation analysis showed that the SASRQ scores significantly and positively correlated with all startle responses, and negatively correlated with the ASAT measurements (Fig. 4).

### EDA

**Fig. 5:**
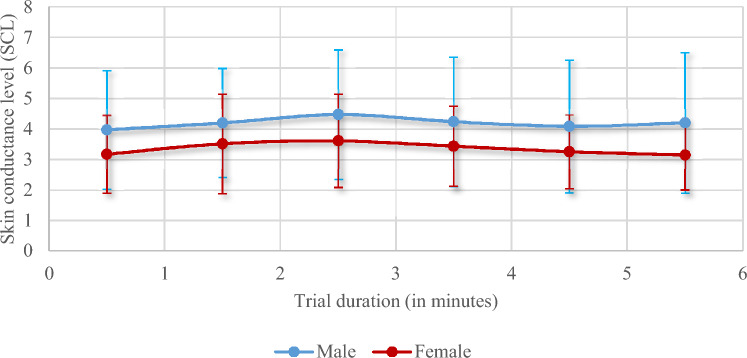
Mean and standard deviation of the skin conductance levels as average per minute of the trial. Both female and male soldiers showed similar and stable conductance levels throughout the auditory trial

The skin conductance levels showed relatively stable levels during the emotional and attentional regulation measurement, with no significant differences between the groups (Fig. 5). The startle-related outcomes (Table 2) show that male soldiers showed a tendency to a significantly higher amplitude of the electro-dermal response compared with the female soldiers (p = 0.029, 95% CI [− 0.328, − 0.018]; corrected alpha level = 0.01).

**Table 2:** EDA skin conductance response-related outcomes

EDA response	Female soldiers (n = 38)	Male soldiers (n = 17)
Average non-specific SCRs per trial minute	Mean = 13.588 (SD 10.575)	Mean = 11.725 (SD 8.259) *p* = *0.529* *95%* *CI* *[−* *3.97,* *7.635]*
Startle amplitude	Mean = 0.334 (SD 0.349)	mean = 0.444 (SD 0.464) *p* = *0.029*^#^ *95%* *CI* *[−* *0.328,* *−* *0.018]*
Rise time	Mean = 3.666 (SD 4.041)	Mean = 3.165 (SD 2.602) *p* = *0.636* *95%* *CI* *[−* *1.624,* *2.637]*
50% decay time	Mean = 90.566 (SD 12.6134)	Mean = 116.588 (SD 137.707) *p* = *0.495* *95%* *CI* *[−* *101.936,* *49.918]*
100% decay time	Mean = 119.987 (SD 128.889)	Mean = 155.824 (SD 135.510) *p* = *0.353* *95%* *CI* *[−* *112.445.* *40.798]*

To correct for Type I errors, the alpha level for a significant difference was adjusted to p < 0.01 using the Bonferroni formula

^**#**^Indicating a result approaching significance

## Discussion

The presented data provides unique evidence for the emotional, attentional, and stress regulation abilities of female and male IDF combat soldiers during a war. We found relatively high SASRQ-scores, indicating high risk for ASR [39], thus concluding that at least some of the combat soldiers are at a risk to develop severe mental health complications, including PTSD [39]. However, as the war is still ongoing and the soldiers are waiting to be deployed, this symptomology needs to be re-assessed after the stressful war situation is over, specifically considering the currently normative resilience scores on both subjective (CD-RISC [40–42]) and objective (startle responses, ASAT [22]) measures.

Considering the high SASRQ scores, and the expected increase in the exposure to war events, the combat soldiers might already be psychologically affected by the war, and therefore could probably benefit from psychoeducation, detection of vulnerable individuals, and a structured transition to civilian life post deployment [43, 44]. In addition, combat soldiers are at risk of being exposed to direct potentially traumatic events, and potentially morally injurious events, including experiencing betrayal, witnessing, or perpetuating an act of moral transgression [45–48]. Attention should be given to this exposure as another risk factor for future mental difficulties, such as alteration to core beliefs and attitudes, depressive symptoms, and chronic stress [49]. As moral transgressions are common in modern warfare [50], especially when fighting in urban locations, against terror organizations that aim to harm innocent people, and among civilian populations [51], the issue of moral injurious events is of high interest to mental health professionals and is specifically relevant in the war against fundamentalist military groups, as is the case in the Israel–Hamas war [48].

Although there was no significant difference between female and male soldiers on the total SASRQ scores, female combat soldiers showed significantly higher hyperarousal scores compared to male soldiers. When self-reported emotional difficulties are concerned, this finding adheres with a significantly larger portion of female soldiers reporting the army service as difficult to adapt to and as an emotional burden.

The neurophysiological data provided possibly different evidence for the relationship between gender and emotion regulation abilities, namely with male combat soldiers showing a tendency to a significantly higher level of physiological arousal (measured by EDA) in response to a sudden startle stimulus. Other than that, there were no significant differences in the EDA nor ASAT scores between the female and male soldiers, indicating similar levels of emotion and attentional regulation abilities, as well as similar levels of arousal and stress.

This almost contradictory finding between the subjective, self-reported hyperarousal scale and the objective, neurophysiological measurements, may provide a unique indication for the way that men and women differ in their interpretation or report their subjective emotional compared with their physiological state. Those gender differences may stem from a combination of biological and social factors that require further research [52, 53], especially as conformity to masculine norms, including limited emotionality, is related to higher rates of PTSD and other mental disorders [54]. With a word of caution, it could mean that female soldiers have lower self-perception, that might compromise their resilience, as previously investigated only in male soldiers [17]. Alternatively, male soldiers might lack emotional awareness [55] and therefore could be at a higher risk of over-assessing their competence, and among other risks, of being under-diagnosed by self-report mental health measures.

The ability to assess acute stress symptoms more adequately with the combined physiological measures is underscored by the significant correlations between the SASRQ and ASAT scores: The higher the self-reported acute stress symptoms, the lower the emotion regulation is (shown by an increased startle response), and the lower the attentional regulation (shown by the reduction in pre-pulse inhibition). Together, this highlights the potential of physiological tools to surpass self-report bias [19, 56]. Those tools can further our understanding of psychological resilience, given the existing concern that subjective reports of resilience might at least partially reflect a ‘self-deception artifact’ [20], or at best a measure of positive self-perception, thus emphasizing the importance of combining self-report and physiological tools aimed to better the prediction of mental health symptoms.

When it comes to differences between male and female soldiers during the war, the total CD-RISC, SASRQ, and ASAT scores showed no significant gender differences. The emotional and attentional regulation abilities are similar despite the unique risks for female soldiers in battle, exemplified by the extreme sexual violence aimed at Israeli women by the Hamas terrorists [57–59] Thus, these finding support previous reports on women’s psychological capability to successfully serve in combat roles [60].

Women—including those serving in the military—face specific gender-related risks. These are highlighted in the event of the October 7 Massacre, as—alongside cases of life-saving insight and heroism from female combat soldiers [61], that in some cases saved dozens of lives [62] there are extensive reports of gender-based violence against female soldiers, including kidnapping, mutilation, and rape [59].

In Israel, despite the involvement of women in combat roles in the independence war of 1948, their assimilation to fighting units was banned until 1985 due to the fear of the enemy using sexual violence against prisoners of war [63], a fear that sadly became reality during the Israel–Hamas war [59, 64]. The strive for gender equality in the military needs to consider both the benefits and the risks inherent in combat service, to the individual, the unit and to society.

A limitation of this research is that the data was taken at one point in time. Data regarding resilience and post-traumatic symptomology may change over time, and those changes are meaningful to the understanding of mental health trajectories [65]. Therefore, there is a need for follow-up measures to estimate the actual resilience of combat soldiers through a longitudinal study design, in which future differential exposure to PTEs and potentially morally injurious events should be considered. In addition, different factors, such as the degree of safety from war that the soldier's experience at home, previous exposure to traumatic events, intelligence, personality [66], and interpersonal [67] or spiritual [68] resilience factors, might impact the results.

Additional limitation is that the sample size is not large (38 females and 17 males), which might contribute to type 2 errors in the comparison between the sexes, in addition to limiting generalizability. Therefore, we would recommend conducting further research on the resilience of male and female soldiers during war, and other individuals under continuous stress, combining self-report and physiological measures, so we can apply wider conclusions to policy, assessment and treatment.

To summarize, our results show that the wartime combat soldiers are hypervigilant but manage to self-regulate their emotional and attentional abilities and thus manage their stress in a normative manner. However, the heightened stress levels and post-traumatic symptomatology could indicate future vulnerability of some of them to develop posttraumatic disorders that should be treated within a critical time frame. Although female soldiers self-reported higher levels of hyperarousal, men showed physiologically increased hyperarousal through the electrodermal startle response. Thus, no significant gender difference was found regarding stress levels, resilience, and ability to self-regulate, underscoring the capabilities of female combat soldiers to maintain allostasis during war, despite being exposed to unique gender-related risks.
